# Ziemssen’s Motor Points of the Human Body

**Published:** 1884-10

**Authors:** 


**Ziemssen's Motor Points of the Human Body.** A Guide to Localized Electrization. By HERBERT TIBBITTS, M. D. New York: J. H. Vail & Co., Medical Publishers, Booksellers and Importers, 21 Astor Place and 142 Eighth Street. Buffalo: J. H. Matteson. 1884.

The profession have, for a long time, needed a guide to local electrization, and the present work furnishes the desired information in a compact and practical form, adapted to the immediate wants of the practitioner. It comprises a brief introduction, in which Ziemssen's electrodes are delineated and explained, and five plates, with ample explanation as to the use of electricity in various parts of the body. We regard the work as a very valuable one, and recommend it to those who seek to attain success in the use of electricity in disease.

